# Effect of Strain on Heating Characteristics of Silicone/CNT Composites

**DOI:** 10.3390/ma14164528

**Published:** 2021-08-12

**Authors:** Minoj Gnanaseelan, Kristin Trommer, Maik Gude, Rafal Stanik, Bartlomiej Przybyszewski, Rafal Kozera, Anna Boczkowska

**Affiliations:** 1 FILK Freiberg Institute gGmbH, Meißner Ring 1-5, 09599 Freiberg, Germany; kristin.trommer@filkfreiberg.de; 2 Institute of Lightweight Engineering and Polymer Technology (ILK), Technische Universität Dresden, 01307 Dresden, Germany; maik.gude@tu-dresden.de (M.G.); rafal.stanik@tu-dresden.de (R.S.); 3 Faculty of Materials Science and Engineering, Warsaw University of Technology, 141 Woloska Str., 02-507 Warsaw, Poland; Bartlomiej.przybyszewski.dokt@pw.edu.pl (B.P.); rafal.kozera@pw.edu.pl (R.K.); anna.boczkowska@pw.edu.pl (A.B.)

**Keywords:** CNT composites, silicone: Joule heating, conductive polymer composites, spread coating, electrical heating

## Abstract

In this work, silicone/carbon nanotube (CNT) composites were produced using a spread coating process, followed by morphological investigations and determination of their electrical properties and heating behaviour through the application of electric potential. Composites containing varying amounts of CNT (1–7%) were investigated for their thermal behaviour with the use of an IR camera. Subsequently, thermal behaviour and electrical properties were measured when the samples were stretched (up to 20%). With the 7% CNT composites, which had a conductivity of 106 S/m, it was possible to achieve a temperature of 155 °C at a relatively low voltage of 23 V. For high CNT contents, when the potential was controlled in such a way as to maintain the temperature well below 100 °C, the temperature remained almost constant at all levels of strain investigated. At higher potentials yielding temperatures around 100 °C and above, stretching had a drastic effect on temperature. These results are critical for designing composites for dynamic applications requiring a material whose properties remain stable under strain.

## 1. Introduction

As many fields of engineering focus on the development of materials that are functional, lightweight, and flexible, conductive polymer composites (CPCs) have attracted interest, as they possess these essential attributes [1]. CPCs are prepared by incorporating electrically conductive particles, such as carbon nanotubes (CNTs), graphene, carbon black, graphite, etc., into an insulating polymer matrix. The polymer matrix can be hard and inflexible, such as epoxy and polymethyl methacrylate (PMMA); tough, such as polyurethane (PU) and polycarbonate; or stretchable, such as silicone, elastomers, and thermoplastic polyurethane (TPU). CPCs have several applications, such as electromagnetic interference (EMI) shielding, antistatic, thermoelectric materials, sensors, actuators, etc. [2,3].

In this paper, CNT-reinforced silicone rubber (SR) composites were investigated. Silicone rubbers are some of the most important materials among inorganic synthetic functional elastomers. They possess unique properties and advantages, such as chemical resistance, thermal stability, low toxicity, and above all high elasticity [4,5]. Because of these properties they are widely used in different sectors, such as for medical devices, implants, sealants, electronics, lubricants, and membranes [6,7]. In practice, SR cannot be used on its own because of its low mechanical properties (tensile strength and Young’s modulus) as well as its low electrical conductivity and thermal stability [8]. Consequently, different fillers are incorporated into an SR matrix to improve its mechanical as well as its functional properties [9,10].

In recent times, carbon allotropes have attracted scientists owing to their ability to improve the thermal stability and electrical conductivity of polymer matrices [11,12]. Among all electrically conductive carbon allotropes, the most preferred types are multiwalled CNTs (MWCNTs) because of their low price, accessibility, and extraordinary properties (such as their high tensile strength) [13], as well as their excellent thermal and electrical conductivity [14,15]. Several polymer composites filled with CNTs, when investigated in recent research, exhibited improved thermal and electrical conductivity. The mechanism of the improvement in properties due to CNTs has been studied [16,17,18]. Unlike other fillers, such as carbon black and graphite, even with a relatively low loading of CNTs into the SR matrix, the mechanical, thermal, or electrical properties of the composites may be significantly improved, making CNTs a promising candidate for a filler. The vastly improved thermal stability of CNT-reinforced plastics has been ascertained in the literature [19,20]. In studies utilising a large amount of CNT as filler, mechanical properties were improved with little deterioration in the inherent properties (e.g., elasticity) of the polymer matrix. For example, CNT/rubber composites show higher thermal stability than that of neat polymer matrix and retain good mechanical properties with only a slight reduction in elongation at break [21]. It has also been proven that the final properties of SR–CNT composites depend not only on their CNT concentration but also on their size (outer diameter, inner diameter, and length) [22]. Apart from mechanical and electrical properties, the electrical heating characteristics of SR–CNT composites have also been investigated previously, but only under static conditions [23,24,25]. As many applications demand stable performance even when the material is stretched, the electrical heating behaviour needs to be analysed also when strained, which has not been reported in earlier works. Hence, the main objective of this work revolves around a comparison of the heating characteristics of several composites under strain.

In this study, CNT/silicone rubber nanocomposites with improved heating behaviour and electrical conductivity were fabricated. In addition to the measurement of static heating characteristics, the effect of strain on heating behaviour as well as on electrical conductivity was investigated.

## 2. Materials and Methods

### 2.1. Chemicals

Liquid silicone rubber (LSR), Elastosil LR 6250 F from Wacker Chemie AG (Munich, Germany) with a viscosity of 100 Pa∙s, shore A hardness of 36, tensile strength of 5 MPa and elongation at break of 350%, was used along with a hydrogen-terminated polysiloxane (Crosslinker W). Multiwalled CNT (MWCNT), NC7000 from Nanocyl^TM^ from Belgium (Sambreville), with an average diameter of 9.5 nm, a length of 1.5 µm and carbon purity of 90%, served as conductive filler. Toluene (99.5% purity) from Roth, and silver powder (99.5% purity) with a mean particle size of 5µm were also used. All the chemicals were used as purchased without any further purification.

### 2.2. Preparation of Silicone/CNT Dispersion

A defined quantity of MWCNT was added to LSR in a container, and the mixture was stirred with a spatula to ensure that the CNT powders were macroscopically mixed. Then, the mixture was introduced into a 3-roll mill, EXAKT 120EH-250, as the rolls rotated. The mixture was sheared through the rolls in 4 discrete passes with decreasing gaps between the rolls. The roll gaps are presented in Table 1.

After the final pass, the dispersion was collected from the last roll in a container. This final step played a dominant role in dispersing CNT in the silicone matrix.

### 2.3. Preparation of Composite Films

The as-prepared dispersion was mixed with 1% crosslinker in relation to the weight of LSR and further diluted with toluene to ensure appropriate viscosity. This step was performed in a 3-roll mill at a gap of 30 µm. The dispersion was then subjected to degassing at a low pressure of 0.8 bar for 30 min.

Composite film was made from this degassed dispersion through a spread coating process. In this process, a polymethyl pentene-coated transfer paper (Schöller, Weißenborn, Germany) was stretched between two thin rubberised rollers in a frame supported by springs. The dispersion was applied to the transfer paper in front of the doctor blade and spread into a thin layer with the help of this blade. The space between the paper and the blade was adjusted with the help of a feeler gauge. A wet film of a defined thickness was formed and then dried at 70 °C for 2 min in an oven to remove remaining solvent. A second coating was then made over the first in order to attain a final film of the desired thickness. It was difficult to attain a film thickness between 150 and 200 µm with only a single coat, as this hindered the escape of the air/vapour bubble from the wet film during the drying stage and created a pinhole when the bubble popped during the curing stage. After the second coat, the film was again dried at 70 °C for 2 min to remove the solvent. Subsequently, the dried film was then cured at 180 °C for 6 min. After removing the composite film from the transfer paper, the thickness was measured. Films with thickness between 150 and 200 µm were prepared.

### 2.4. Application of Contacting Electrodes

In order to apply electrical power to the composite film, the film needed to be electrically contacted by means of a highly conductive coating to ensure reduced power loss at the point of contact. The highly conductive coating was realised using a highly filled silicone dispersion based on silver microparticles. Silver microparticles were mixed with silicone in the presence of a coupling agent. A small amount of toluene was added to aid easy mixing and provide a paste like consistency so that it could be sieve-printed over the composite films. A template made of Teflon (4 mm × 300 mm) was used for printing. Approximately 3 g of the paste was spread out over the template by means of a small screen printing squeegee. Two electrodes were printed on the composite surface at a distance of 5 cm. The printed films were subsequently dried at 100 °C for 2 min to remove the solvents and then cured at 160 °C for 4 min.

### 2.5. Morphological Characterisation

Cross-sections of the samples were made with a sharp razor blade. The cross-sections were then coated in a very thin layer (3 nm) of gold and observed using a scanning electron microscope, Quanta 250 FEG (FEI, Dresden, Germany). A secondary electron detector (SE2) was used for imaging at an operating voltage of 10 kV with a working distance of approximately 10 mm.

### 2.6. Electrical Conductivity Measurements

Electrical conductivity was measured with Loresta equipment (Mitsubishi Chemical Analytech Co., Ltd, Japan) using a 4-point method, where 4 electrodes were placed over the composite film. A defined current was applied through the outer electrodes, and voltage drop was measured by the inner electrodes. The conductivity was measured on a composite film (mentioned in Section 2.3) approximately 30 cm × 30 cm in size, with a thickness of between 150 and 200 µm. The conductivity was measured after conditioning the samples for 24 h at 23 °C and 50% relative humidity.

### 2.7. Heating Behaviour Measurements

The electrically contacted films were cut to a size of 10 cm × 10 cm in which the contacted area was 5 cm × 10 cm. The sample was supported over a stretchable frame by clamping it in rubberised rollers so that the sample did not rest over the table, and there was an air gap in between to avoid conductive heat loss. Electrical power was supplied through a multimeter, with the help of which voltages between 6 and 60 V were realised in steps of 6 or 12 V. Simultaneously, the current flowing through the sample was measured in the multimeter. The temperature distribution over the sample was measured using an IR camera and IRIS plus software. The mean of the temperature distribution was then used for further analysis.

### 2.8. Heating Behaviour Measurements While Stretching

**Figure 1:**
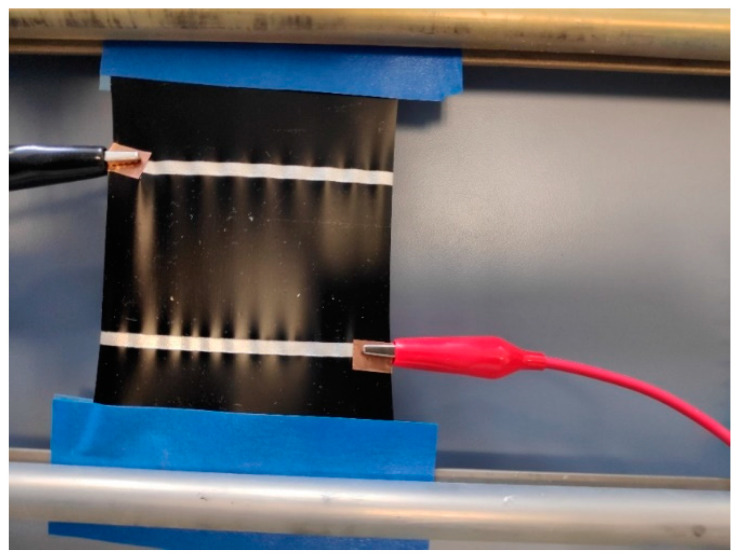
Sample mounted on a stretchable frame.

The sample as mentioned in Section 2.7 was also used here. One of the rubberised rollers could be moved, so that the sample stretched (Figure 1). As it was stretched, the rubberised rollers could be clamped using a screw. In this manner, the samples were stretched by 5%, 10%, 15% and 20% of their initial dimension. After stretching, the same procedure was followed as mentioned in Section 2.6 to measure the heating behaviour at every stage of stretching. After each stage of stretching, the sample was released and allowed to relax for at least 2 h.

## 3. Results and Discussion

### 3.1. Morphology of Silicone Composites

**Figure 2:**
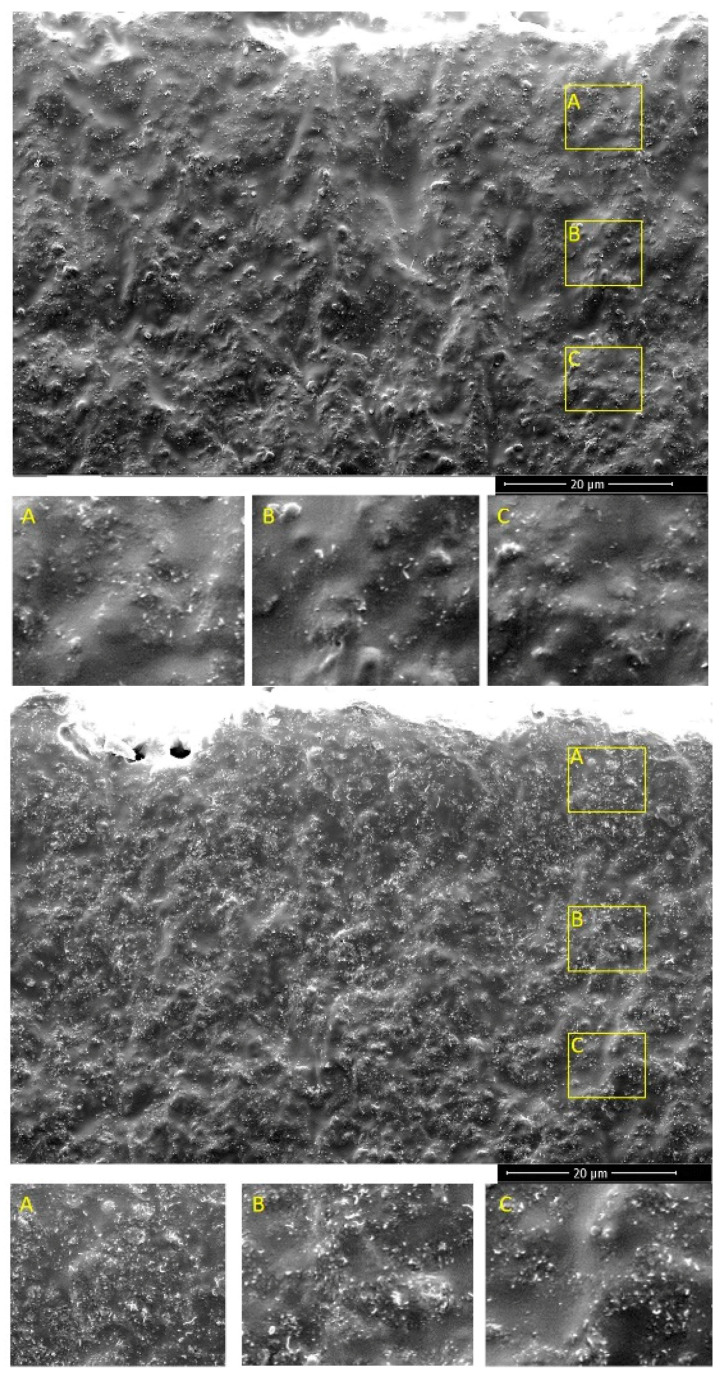
SEM images of silicone composites with 1% CNT (top) and 5% CNT (bottom).

The morphology of the silicone composites was investigated by SEM observation. In Figure 2, it can be observed that the CNT aggregates were distributed homogeneously throughout the samples, irrespective of the CNT concentration. This was analogous to the observations of Chu et al. [23]. CNTs are purchased in large agglomerates. After 3-roll milling in the presence of polymer, the agglomerates break down into small aggregates, which are responsible for the formation of a conductive network. Figure 2 (top) shows silicone composites with 1% CNT, a relatively low content of CNT as evidenced by the lower quantity of white spots (CNT) on the cross-section. In the case of composites with 5% CNT (bottom), the increase in the number of CNT aggregates is clearly visible. Although the CNT loading was higher, no large agglomerates were observed. This indicated that the CNTs interacted well with the polymer matrix.

### 3.2. Electrical Property of Silicone Composites

**Figure 3:**
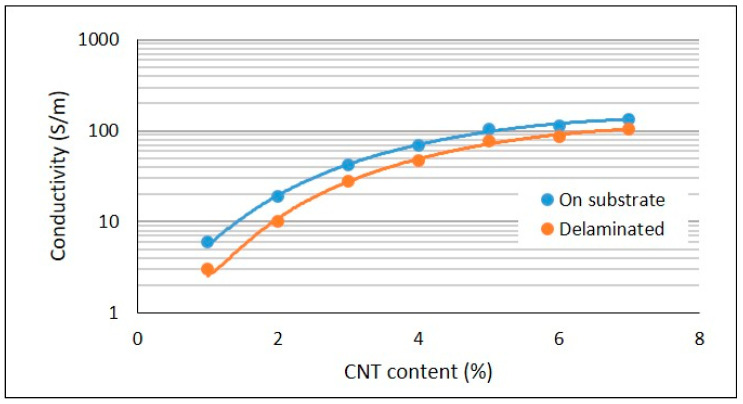
Electrical conductivity of silicone/CNT composites in relation to filler content.

The electrical conductivities of the silicone composite films are presented below in Figure 3.

The blue curve represents the electrical conductivity of the composites that were supported by substrate, while the orange curve represents the electrical conductivity of the composites that were delaminated from the substrate. As expected, the electrical conductivity of the composites increased with CNT loading. As the CNTs were added to the silicone matrix, at a loading of 1%, the CNT aggregates came into contact with each other, forming a continuous conductive network, allowing electron flow throughout the sample. It was not possible to measure the electrical conductivity of composites with CNT loading of 0.1% and 0.5%, as the conductivity was very low, indicating that percolation occurred at approximately 1% CNT, which is also seen in other works [24,25]. As the CNT content was further raised, the conductive network became denser and provided more pathways for the electrons to flow, thereby leading to an increase in electrical conductivity. It was also observed that while raising CNT content to 3% led to a substantial increase in electrical conductivity, but when the CNT loading further rose to 5%, the upsurge in electrical conductivity was not high. Upon further increase to 7%, conductivity increased by only a marginal increment, yielding a conductivity of 106 S/m. Chu et al. also attained similar values with silicone/MWCNT composites [26,27]. It was observed that the conductivity of the composites did not remain the same after delamination. When the composite was prepared on the substrate, the polymer molecules were in a stressed state. Upon delamination, the molecules tended to move towards an entropically favourable configuration, a coiled state. As the polymer coiled, the CNT aggregates were pushed further from each other, leading to a decrease in the contribution from CNT non-ohmic contacts (nanotubes in the contact were separated by several polymer chains) in the conductive network. As there was only a slight decrease in conductivity, it was inferred that there was no decrease in contribution from ohmic contacts (direct contacts between the nanotubes) [28]. Such a decrease in conductivity on delamination was observed at all concentrations but was more evident at very low concentrations (<2%). Composites ideal for heating applications have a conductivity greater than 10 S/m, limited by considerations concerning safety during operation [29].

**Figure 4:**
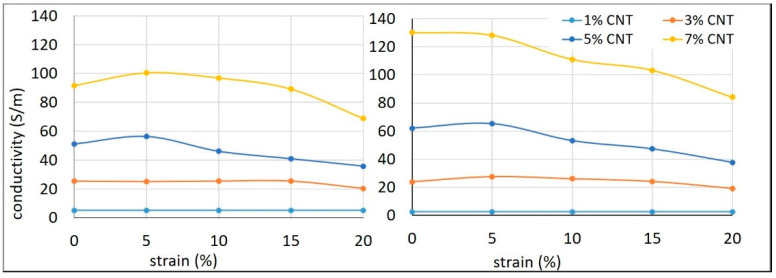
Effect of strain on the conductivity of silicone composites at different CNT concentrations at 6 V (**left**) and 24 V (**right**).

Trends observed in electrical conductivity in relation to strain for silicone composites with varying CNT contents are presented in Figure 4 at four different voltages. It was initially thought that 1% CNT composite would lose its conductivity rapidly when stretched. Contrary to the expectations, for 1% CNT composites, no relationship was observed between conductivity and level of strain. There was no significant loss of density in the conductive network until stretching reached 20%. A similar phenomenon was observed for 3% CNT composites, with only a slight variation. Such behaviour could be due to a greater length of CNT (1.5 µm), while composites with shorter CNTs (0.8 µm) lost their conductivity even at low strains [30]. In the case of the 5% CNT composite, conductivity remained almost constant up to a strain of 5% and then began decreasing considerably. It can be inferred that the conductive network starts disintegrating from a strain of 10%. A similar phenomenon was observed with the 7% CNT composite, wherein conductivity remained constant up to a strain of 5%, and then decreased rapidly. The effect of voltage on the conductivity of composites with lower CNT content was negligible, but at higher CNT contents it was pronounced, especially at 7% CNT. Generally, at higher voltages, more charge carriers are created, which should result in higher conductivity. However, in lower CNT content composites, the number of CNT contacts was very low, acting as a bottleneck for electron flow. Conversely, for composites with higher CNT contents there were numerous CNT contacts; hence, the charge carriers were not interrupted and contributed to the increase in conductivity.

### 3.3. Heating Behaviour

**Figure 5:**
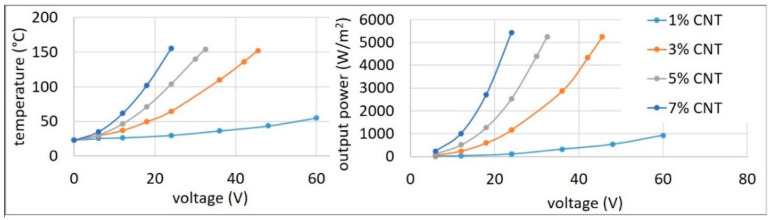
Increase in temperature (**left**) and output power (**right**) with voltage at different CNT concentrations.

The heating behaviour is shown in Figure 5. As electrons flow through the CNT, heat is developed as a consequence of the Joule effect.

**Figure 6:**
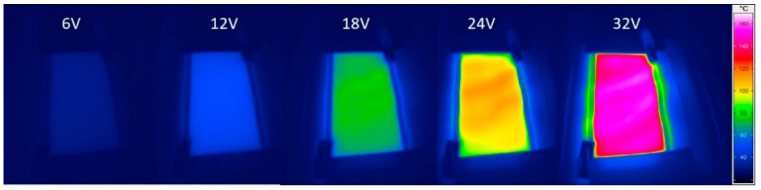
IR images of silicone/5% CNT composite with 5 cm electrode distance at different potential.

Composites with a CNT content of between 1% and 7% were investigated. An example of how the temperature was measured is shown in Figure 6. At 1% loading, the CNTs were already percolated; hence, the composite experienced joule heating upon the application of electrical potential. It was observed that at 1% CNT content, the temperature increase was almost linear with the increase in voltage. The maximum temperature achievable for the 1% CNT composite, which had a conductivity of 3 S/m, was 55 °C at 60 V. There was a marked difference in the heating behaviour for 3% CNT compared to 1% CNT. This was due to almost an order of increase in conductivity (28 S/m) with respect to the 1% CNT composite [31]. The 3% CNT composite reached a temperature of 150 °C at a relatively low voltage (45 V). This temperature was approximately four times higher than that for the 1% composite. It was also able to attain higher temperatures, but to avoid thermal degradation of the composite, the maximum temperature was limited to below 160 °C. Moreover, in contrast to the linear correspondence of temperature to voltage in the case of the 1% composite, an exponential increase was observed. In the case of the 5% CNT composite, the temperature rise was rapid and reached a temperature of 155 °C at a lower voltage of 33 V. In the case of the 7% CNT composite, with an electrical conductivity of 106 S/m, a steep increase in temperature was observed, rising to 155 °C at a very low voltage of 23 V.

### 3.4. Heating Behaviour When Uniaxially Strained

The heating behaviour of silicone composites was investigated when the samples were strained, as this relationship is relevant in most heating applications. The heating behaviour of the 1% CNT composite was represented in two formats. The plots on the left show the variations with voltage and the plots on the right with strain.

**Figure 7:**
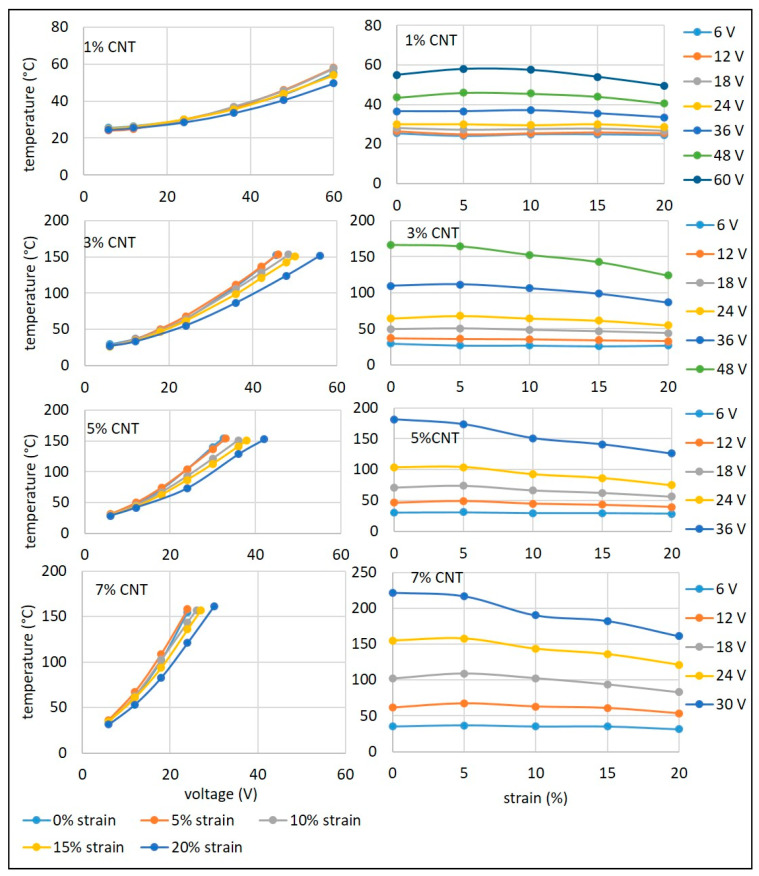
Increase in temperature with voltage (**left**) and with strain (**right**) at different CNT contents.

For each sample, the composite was stretched to four different strain levels (5%, 10%, 15%, and 20%) in a direction perpendicular to the electrodes. As the sample was elongated, the connections between CNTs were reduced, and the conductive network became loose, resulting in a fall-off in conductivity. In Figure 7, it can be observed that the heating behaviour of the composite up to 24 V was almost constant at all the strain levels. The same applies to the observations of Figure 4, which states that the conductivity of the 1% CNT composite remained almost constant at all strain levels up to a potential of 24 V. Above 24 V, heating behaviour when strained up to 10% was almost the same, with very slight differences at higher voltages. However, heating behaviour at 20% strain was considerably lower than at other strain levels. While at 10% strain or less, there was little change in heating behaviour, at 20% strain, there was a considerable difference, which can be attributed to the fact that the CNTs had moved apart considerably and the number of contacts were reduced, leading to fewer pathways for electron flow.

In comparison to the 1% CNT composite, the temperatures achievable at different strains were much higher for the 3% CNT composite (Figure 7). This was primarily due to the denser conductive network being affected by the higher CNT loading, thereby creating more pathways for electrons to produce Joule heating [32]. As observed for the 1% CNT composite, heating behaviour remained the same up to 24 V at all strain levels. However, at higher potential, though higher temperatures were reached, when the composite was strained to 10%, the temperature started to deteriorate; at 15% strain, there was further decrease; and at 20% strain, there was a significant difference. 

In comparison to the 3% CNT composite, higher temperatures were achieved at much lower voltages for the 5% CNT composite at all strain levels (Figure 7). As observed for the 3% CNT composite, heating behaviour remained the same up to 12 V at all strain levels. However, at 18 V, there was a slight decrease in temperatures at higher strains. At 24 V, the temperature remained constant up to a strain of 5%; when strained to 10%, the temperature started to deteriorate; at 15% strain, there was a further decrease; and at 20% strain, there was a considerable difference. At 36 V, the temperature started to decrease immediately from a strain of 5% and then decreased extensively at higher levels of strain.

In comparison to the 5% CNT composite, higher temperatures were achieved at relatively low voltages for the 7% CNT composite at all strain levels (Figure 7). As observed for the 3% CNT composite, the heating behaviour remained the same up to 12 V at all strain levels. However, at 18 V the temperature was almost constant up to 10% strain, and then there was slight decrease in temperature at higher strains. At higher voltages (24 and 30 V) up to a level of 5% strain, the temperature was constant, and then it gradually decreased.

## 4. Conclusions

The delicate but efficient mixing method using a 3-roll mill yielded silicone/CNT composites up to a loading of 7% CNT, with good filler dispersion even at such a high content. Good dispersion quality was reflected in the electrical conductivity achieved, with a conductivity of 106 S/m attained at 7% CNT content. Interestingly, composites with lower filler content (1% and 3%) exhibited conductivity independent of strain level owing to the lower density of the conductive network. On the contrary, composites with higher filler content (5% and 7%) displayed strong strain-dependent conductivity, especially at strain levels above 10%. Such behaviour can be attributed to the loosening of the denser conductive network. The sharp rise in temperature for composites with higher CNT content was by virtue of their higher conductivity, whereby a 7% CNT composite was able to achieve a temperature of 155 °C at a lower voltage of 23 V. In the case of composites containing lower CNT content (1% and 3%), only at very high voltages (from 36 V) did strain level affect the rise in temperature. Conversely, for composites containing higher CNT content (5% and 7%), the influence of strain level started at lower voltages (from 18 V). Such behaviour can again be attributed to the density of the conductive network, in which at lower filler contents, the change in conductive network structure is lesser than at higher filler contents. For applications demanding very little temperature change, it is advisable to select composites with lower CNT content, while for applications that demand lower input voltage, composites with higher CNT content would be the wiser choice.

## Figures and Tables

**Table 1:** Gap between the rolls set in the 3-roll mixing process.

Sequence	1st Gap (µm)	2nd Gap (µm)
1st pass	150	50
2nd pass	40	20
3rd pass	15	10
4th pass	5	5

## Data Availability

The data presented in this study are available on request from the corresponding author.
